# Metabolic Effects of Access to Sucrose Drink in Female Rats and Transmission of Some Effects to Their Offspring

**DOI:** 10.1371/journal.pone.0131107

**Published:** 2015-07-02

**Authors:** Michael D. Kendig, Winda Ekayanti, Hayden Stewart, Robert A. Boakes, Kieron Rooney

**Affiliations:** 1 School of Psychology (A18), University of Sydney, Sydney, New South Wales 2006, Australia; 2 Human Nutrition Unit, School of Molecular Bioscience, Building G08, University of Sydney, Sydney, New South Wales 2006, Australia; 3 Exercise, Health and Performance, Faculty of Health Sciences (C42), Cumberland Campus, University of Sydney, Lidcombe, New South Wales 2142, Australia; 4 Charles Perkins Centre, University of Sydney, Sydney, New South Wales 2006, Australia; Barnard College, Columbia University, UNITED STATES

## Abstract

The aims of this study were, first, to examine the metabolic consequences for female rats of having unrestricted access to 10% sucrose solution and, second, to test for effects of this dietary intervention on their offspring. In Stage 1 females were mated following a 4-week period in which one group was given the sucrose in addition to their normal chow and a control group was given chow and water only. Sucrose was removed at parturition and the pups monitored until weaning. Despite the development of glucose intolerance in sucrose-fed mothers, no effects were detected on litter size or pup weights. In Stage 2 voluntary activity of offspring was assessed over postnatal days (PND) 51-60 and their glucose tolerance measured at PND89-94. Again no effect of maternal diet was detected. Only male offspring were used in Stage 3, which began when they were 13 weeks old. Four groups were given 10% sucrose solution for 48 days in a 2 x 2 design, in which one factor was maternal diet and the other was whether they were given 2-h access to an activity wheel on alternate days. Higher fasting glucose levels were found in offspring of sugar-fed mothers. Exercise increased insulin sensitivity in these rats but not in offspring of control mothers. Behavioural measures of memory in Stage 3 did not reveal any effects of maternal diet or exercise. Overall, this study suggested that, while providing 10% sucrose solution *ad-libitum* was sufficient to impair maternal metabolism, the impact of this dietary manipulation on offspring may be revealed only when the offspring’s diet is similarly manipulated.

## Introduction

Human epidemiological [1] and experimental studies [2] as summarized in systematic reviews and meta-analyses [3,4] report a link between sugar-sweetened beverage (SSB) consumption and chronic diseases such as obesity, type 2 diabetes and the metabolic syndrome. This evidence from human studies is consistent with that from animal research. Several studies have reported long-term SSB consumption by rats to result in the development of fatty liver, elevated abdominal adiposity, and impaired glucose tolerance [5–9]. However, in most of these studies only males have been investigated. Potential sex differences are of interest for at least two reasons. First, human evidence indicating sex differences in acute metabolic responses to fructose consumption suggests that females may be somewhat protected against fructose-induced hypertriglyceridemia [10]. Second, SSB consumption has been reported to exacerbate a genetic predisposition to obesity [11] and this raises the question of whether maternal sugar intake can affect offspring development.

Dietary insults to the intrauterine environment are associated with altered fetal development and subsequent progressive metabolic disorders in adulthood. In both human and small animal studies maternal over-nutrition has been associated with offspring obesity [12]. While many small animal studies have investigated the effects of maternal diets high in both sugar and fat, or high in fat alone [13, 14], few have isolated the effects of diets high in sugar alone on offspring’s metabolic development.

Early studies investigating the effects of maternal sugar intake provided sugar (sucrose or fructose) in high concentrations (50–65% of total energy) as part of a solid chow diet, with access commencing usually at the time of gestation [15] or within 2 weeks of mating [16], although in one study for up to 90 days prior to mating [17]. The strongest evidence of an effect of maternal sugar intake on offspring development came from a study that used a solid diet with high sugar content and reported elevated blood glucose and triglyceride levels in the 100-day old, chow-fed offspring of sucrose-fed mothers [18]. Effects in offspring have also been shown prior to parturition: One study reported that 15- or 19-day old fetuses of mothers fed sucrose starting at pregnancy day 1 exhibited poorer hepatic lipid and glycogen metabolism relative to offspring of mothers fed dextrin [19].

More closely related to the present study are three experiments in which pregnant rats were provided with access to a sugar solution as an addition to their normal chow diet. In the first, pregnant rats were given unrestricted access to either 10% sucrose solution or 16% high-fructose corn syrup (HFCS) solution (or water control) from embryonic day 5 through to parturition, with no weight differences detected between these groups [20]. Among the offspring that were fed only chow and water, those from sugar-fed mothers weighed more than offspring of control mothers at 4 months of age and exhibited enhanced locomotor sensitivity to amphetamine at 6 months of age. In the second study female rats were provided with access to either a 10% fructose or 10% glucose solution (or water control) from the first day of conception through to day 21 of pregnancy [21]. Fetuses collected on pregnancy day 21 of fructose-fed mothers displayed reduced blood triglycerides but elevated liver triglycerides compared to glucose supplemented and control mothers. In the third study, female rats were given free access to 20% sucrose solution from gestational day 1–21 in addition to regular chow [22]. Male adult offspring from sucrose-fed mothers were heavier at 1- and 2-months of age; showed poorer performance on the Morris Water Maze, a hippocampal-dependent measure of spatial memory, and exhibited hippocampal dysfunction on a range of cellular markers.

To the extent that these studies serve as a model of human nutrition, introducing sugar solutions at the start or early in pregnancy is arguably less useful than making sugar solutions available long before the females become pregnant. Consequently the present study adopted the latter approach, in that female rats were given the sugar solution for 4 weeks before being mated. For a similar reason the sugar used here was sucrose at a concentration of 10%, since this closely approximates the sweetener used in most commercially available beverages outside of North America. Finally, access to sucrose in the present study was terminated at parturition so as to limit any effect of maternal transmission of sucrose via lactation. Among previous studies of the present kind it appears that only one [22] has taken this approach.

In our previous studies using male rats, *ad-libitum* access to 10% sucrose solution has consistently resulted in elevated adiposity and impaired glucose function [9, 23–25] but we have not previously tested its effect in females. The present study employed three stages to address the following aims.

The aim of Stage 1 was to investigate the effect of unrestricted access to both 10% sucrose solution and water on the metabolic health of female rats prior to mating and during pregnancy and on the early postnatal development of their offspring. The aim of Stage 2 was to examine the effect of maternal sucrose consumption on the glucose tolerance and physical activity of young adult offspring when fed only chow and water. The aim of Stage 3 was to examine the effect of providing the now-adult offspring with unrestricted access to 10% sucrose solution, with or without exercise, on metabolic outcomes and on spatial and non-spatial memory.

## Methods

### 2.1 Animals

Forty-eight (24 male and 24 female) outbred albino Wistar rats (*Rattus norvegicus*) were purchased from the University of Adelaide. They were 8–9 weeks old at the start of the study when they were separated by sex into groups of 8 for initial housing (3 male and 3 female cages) and for feeding during 9 days of acclimatization. Large plastic cages with steel bar lids (58 x 31 x 36 cm) were used for group housing and small cages (44 x 28 x 29 cm) were used for individual housing and breeding. The colony room was maintained at 23–25°C with humidity at 40–45%. A 12:12 hour reverse dark-light cycle (lights on at 21:00) was employed throughout the experiment. All rats had unrestricted access to standard chow (Specialty Feeds, 14.2kJ/g; 20% protein, 4% fat, 60% carbohydrate) and tap water throughout the study. Water and sucrose solutions were available from 500-ml plastic bottles with metal ball-bearing spouts inserted into the animals’ cages. The 10% (^w^/_v_) sucrose (Coles white sugar; Victoria, Australia) solutions were made with tap water.

### 2.2 Apparatus

The activity tests and intervention used in Stages 2 and 3 used 10 custom-built activity wheels with removable plastic cages. The plastic cages measured 33 x 21 x 19 cm and had a mesh lid with attachments for water bottles and food access. Cages were placed next to the wheels so openings in both the cage and wheel lined up allowing wheel access. The wheels were 10 cm wide and 1.1 m in circumference. Access from the attached cage could be prevented by sliding doors. Wheel revolutions were stored in 30-s bins using Labview software.

The recognition tests were conducted in an open-field arena (60 x 60 x 50 cm) constructed from black PVC plastic and plywood. A variety of commercial products (e.g., sauce bottles and small plastic containers) of varying materials (glass, plastic and porcelain) were used as objects. Three copies of each object were used so that each object was only used for one trial per rat and to prevent odours from previous contacts serving as cues. A 50% ethanol solution was used to clean and dry the arena between each trial. A video camera positioned above the arena was used to record behaviour, which was later scored using Macropod ODLog 2.x software by condition-blind observers.

### 2.3 Procedures

#### 2.3.1 Stage 1: The effect of unrestricted access to 10% sucrose solution on the metabolic health of female rats prior to and during pregnancy and on the early postnatal development of their offspring

A timeline of all experimental procedures is shown in Table 1. For Stage 1 rats were re-housed into groups of 4 for the initial 4 weeks of the dietary intervention. Half of the female rats (Sucrose group: *n* = 12) received unrestricted access to 10% sucrose solution and water in addition to chow, while the Control group (*n* = 12) received only water and chow. The 24 males were maintained on chow and water during these 4 weeks. Female body weights and their chow were weighed every 6 days and their drinking bottles (water and sucrose) were weighed daily. Males were also weighed every six days.

**Table 1:** Timeline of experimental procedures.

**Stage 1 –Maternal Sucrose**					
		♂ *N* = 24 Control	**♀** *n* = 12 Sucrose		**♀** *n* **=** 12 Control
**Diet** (4 wks)		Chow & water	Chow, water, 10% suc		Chow & water
**Mating** (1 wk)		Pair-housed with **♀**	Chow, water, 10% suc		Chow & water
**Gestation** (3 wks)		Removed from study	11 pregnant (chow, water, 10% suc)		11 pregnant (chow & water)
**Parturition** (PND 1–21)			121 offspring (chow & water)		133 offspring (chow & water)
**Weaning** (PND 21)		Litters reduced to 5:3 ♂:**♀**.; fed chow and water; housed by sex, condition (*n* = 5-8/cage). Mothers culled			
Offspring (*N* = 164): 48 suc ♂, 50 con ♂, 32 suc ♀, 34 con ♀					
For Stage 2 (*n* = 40)		For Stage 3 (*n* = 48)	For other experiments (*n* = 76)		
**Stage 2 –Young adult offspring**					
***N* = 40**					
		Con Female	Con Male	Suc Female	Suc Male
**Wheel running** (PND 50–60)	*N* = 40	*n* = 10	*n* = 10	*n* = 10	*n* = 10
**OGTT** (PND 89–94)	*n* = 28 (subset)	*n* = 7	*n* = 7	*n* = 7	*n* = 7
**Stage 3 –Sucrose and activity intervention**					
***N* = 48 ♂ offspring (PND 91–94)**					
	*n* = 20 Control offspring		*n* = 28 Sucrose offspring		
	*Con-Sedentary*(*n* = 10)	*Con-Active* (*n = 10)*	*Suc-Sedentary* (*n* = 10)	*Suc-Active* (*n* = 10)	*Added* (*n* = 8)
**Cognitive test**	12 days				Chow and water in home cage
**Sucrose & Activity**	20 days; 10% sucrose (all); activity (Con-Active & Suc-Active)				
**Fasting glucose**	1 day (after 12-h fast)				
**Cognitive test**	8 days; 10% sucrose (all); no activity				
**Sucrose & activity**	20 days; 10% sucrose (all); activity (Con-Active & Suc-Active)				
**Fasting glucose**	1 day (after 12-h fast)				
**Cull**	Over 2 days (PND 151–154)				

On Day 29, the females were deprived of food overnight before testing for blood glucose and lipids. Fasting blood triglyceride content was measured with a triglyceride-specific test strip for a point-of-care blood analyzer (CardioCheck Analyzer, PTS, Inc.). Blood glucose levels at fasting and during a subsequent oral glucose tolerance test (OGTT) were measured using a diagnostic glucometer (OneTouch Verio, Johnson & Johnson), with blood samples obtained from the tail after removing the tip with a sterile scalpel. An additional 150μL of blood was diluted in 50μL saline, centrifuged at 10,000rpm and at 4°C for 20 min. Plasma was then extracted and stored at -80°C until analysis. Following baseline fasting assessment, animals received a 3g/kg body weight dose of a 50% (w/v) glucose solution via oral gavage and blood glucose level (BGL) was measured at 20, 40, 60, 90 and 120 min post-gavage.

On Day 31, male and female rats were randomly paired for mating. Each pair was kept in a small cage for 7 days, after which the male rats were returned to group housing and later made available for other studies in the School of Psychology. Pairs containing a female from the Sucrose group had continued access to 10% sucrose throughout mating. When a female’s weight during this period indicated that she had not become pregnant, she was re-mated with a different male. While male rats paired with a Sucrose female could also consume sucrose during this week, it appears unlikely that this brief duration of exposure would be sufficient to produce metabolic effects in these male rats. Sucrose access was also continued for the females during the 21-day gestation period, when they were weighed every 2 days. As soon as a birth occurred to a sucrose mother, the sucrose solution was removed from the cage.

Within 24 h after a birth occurred, litter size, sex and weight of each pup were recorded. Sex was identified by inspection of the genitals. Litters were then reduced to 8 offspring, with an optimal ratio of 5 males to 3 females, but a ratio of 4:4 was used when there were more females than males in the original litter. The ratio 5:3 was preferred as only males were to be used in Stage 3. Offspring were selected based on median body weight (exclusion of the lightest and heaviest offspring, then the second lightest and heaviest offspring and so on). Excluded pups were culled by cervical decapitation. During the 21-day lactation period pups were weighed at days 1, 4, 7, 10, 13, 16, 19 and 21.

Weaning occurred at post-natal day (PND) 21. At this time pups were removed from their mothers and group-housed, separated by sex, with free access to chow and water. At weaning mothers were weighed, culled by intraperitoneal injection of sodium pentobarbital and retroperitoneal fat pads were excised and weighed.

In total, 254 pups were successfully delivered from a total of 22 litters. Of these, 164 were retained for the tests described below; these consisted of 80 offspring from sucrose mothers (48 males, 32 females) and 84 from control mothers (50 males, 34 females). At PND 50, one offspring from each litter was randomly allocated to one of three groups. Group 1 consisted of 40 rats (10 males and 10 females from control mothers and 10 males and 10 females from sucrose mothers) that completed Stage 2 below. Group 2 consisted of 48 males: 20 originating from 9 litters of control mothers and 28 originating from 10 litters of sucrose mothers. These males were used in Stage 3. The remaining 76 animals (Group 3) were made available to other studies within the School of Psychology and School of Physiology. Mean body weights at this time were 277 g ± 4.8 g (SEM) for males and 219 ± 3.2 g for females in Group 1; 269 g ± 3.7 g for males in Group 2; and 230 ± 9.6 g for the males and 218 ± 4.8 g for the females in Group 3.

#### 2.3.2 Stage 2: Glucose tolerance and physical activity in young adult offspring

Stage 2 examined whether maternal sucrose consumption would affect glucose tolerance and voluntary activity levels of offspring that were fed only chow and water. Possible effects of maternal diet here were important to identify prior to the commencement of Stage 3, which involved a diet and exercise intervention. Throughout this stage the 40 rats of Group 1 (see above) remained group-housed (*n* = 4-6/cage) with free access to chow and water, and were weighed weekly.

Voluntary exercise was assessed in three 1-h wheel running sessions held on alternate days during PND 50–60 between 1300 and 1500 h. Since one pair of male control offspring and one pair of female control offspring originated from the same litters, data were averaged for each pair so that male and female Control groups each had *n* = 9 in this analysis.

At PND89-94, an OGTT was administered to a random sample of 14 female offspring and 14 males (7 from Sucrose and 7 from Control mothers for each sex) from Group 1. The OGTT followed a 12-h fast during the animals’ light phase and involved the same procedure as that described for Stage 1. Because two pairs of female control offspring originated from the same litter, blood glucose values were averaged for each pair at each time point so that their litter was represented once only. Finally, one sucrose female could not be gavaged and so was excluded. Consequently, for this analysis there were 7 male and effectively 5 female offspring from control mothers and 7 male and effectively 6 female offspring from sucrose-fed mothers.

#### 2.3.3 Stage 3: The effect of sucrose consumption with or without exercise on metabolic and behavioural outcomes in the adult male offspring of sucrose-fed mothers

At the start of this stage these 48 male rats (Group 2) weighed on average 492 g (401–584 g) and were between PND91-94. Only male rats were used due to limited resources and so as to maintain adequate statistical power to detect effects. Forty of these rats were given unrestricted access to standard chow and 10% sucrose solution. Ten offspring each from sucrose-fed and control mothers were assigned to either Active or Sedentary conditions that were matched for body weight. The resultant groups were labeled: Sucrose-Active; Sucrose-Sedentary; Control-Active; and Control-Sedentary, where ‘Sucrose’ and ‘Control’ indicated their mothers’ diets. Rats were group-housed in condition-matched cages (5/cage). The remaining eight rats were an Added group: offspring of sucrose-fed mothers that received only chow and water with no activity manipulation. This control group allowed for the evaluation of the metabolic effects of sucrose and exercise in the other offspring of sucrose-fed mothers (Sucrose-Active and Sucrose-Sedentary groups).

Prior to the sucrose and activity intervention, the 4 main groups underwent a 12-day baseline measure of object and location recognition, as previously described [24, 26]. Briefly, over 4 days rats were tested for object and location recognition memory in a counterbalanced sequence. Test sessions began with a 5-min familiarization phase in which rats were placed in the test arena with two identical objects placed diagonally opposite in two of the middle four squares of the 16-square grid. Following a 5-min retention phase where a rat remained in its home cage, in the 3-min test phase one of the original objects was moved to a novel location (place task) or was replaced with a novel object (object task). The key measure of memory was the Exploration Ratio, defined as the proportion of object exploration time in the test phase spent exploring the novel object or object in a novel location. Higher ratios reflect greater relative exploration of the novel (or novel-placed) object and better short-term object or location recognition memory.

The 48-day sucrose and activity intervention for the four main groups commenced immediately after baseline behavioural testing. For the duration of the intervention rats received free access to 10% sucrose solution in addition to chow and water, except prior to blood glucose measurement, as described below. Chow and drink bottles were weighed and replenished every 2 days. Exercise training for the Sucrose-Active and Control-Active groups also began immediately after the baseline recognition tests and continued on alternate days, except for the 8-day period when the recognition tests were repeated, as described below. Exercise was voluntary and the procedures and apparatus were identical to those described for Stage 2, except that sessions lasted 2-h rather than 1-h. Two ‘squads’ of rats (each containing 5 rats from Sucrose-Active and Control-Active groups) received their 2-h sessions from 09:00–11:00 h and 11:10–13:10 h. On exercise days the Sedentary groups were transported to the activity lab and handled at the same time as the Active groups, but otherwise remained in their home cages. All 40 of these rats, and the 8 rats in the Added group, were weighed at Day 0 of the intervention and thereafter every 6 days.

For the four main groups the recognition tests were repeated on Days 22–29 of the intervention using the same procedure as at baseline. During this time sucrose access continued but exercise training was suspended and resumed on Day 30. On Days 21 and 51 fasting BGLs were measured in the four main groups following a 12-h fast and using the same procedures as in Stage 1, and the QUICKI index of insulin sensitivity was calculated (QUICKI = 1/(log[Gluc] + log[Ins])). These measures were assessed in the Added group on Day 51. Following fasting blood measurement on Day 51, all 48 rats were maintained on free chow and water for 48 h and were then euthanased on PND151-154 by intraperitoneal injection of sodium pentobarbitone (Lethabarb, Virbac (Australia) Pty, Ltd) for post-mortem dissection of retroperitoneal fat pad mass.

### 2.4 Ethics Statement

All experimental procedures were approved by the University of Sydney Animal Ethics Committee, in accordance with the Australian NHMRC *Animal Code of Practice for the Care and Use of Animals for Scientific Purposes* (Protocol L29/8-2010/3/5354).

### 2.5 Data Analysis

Statistical analysis was performed using SPSS (IBM SPSS Statistics 21, New York, IBM Corp.). The criterion for significance was set at *p* < 0.05. Trends over days in body weight, fluid and chow intakes, as well as blood glucose curves during OGTTs, were analyzed using repeated measures ANOVA. Maternal weights during the diet intervention, gestation and lactation were analyzed separately, with weight data for the latter two phases entered relative to when each rat delivered a litter. For between-group comparisons in Stage 1, Sucrose and Control females were compared using independent samples *t*-tests. In Stage 2 wheel revolutions across days and glucose tolerance were each assessed in mixed-model ANOVAs. For Stage 3, data from the four experimental groups were analyzed in 2 x 2 ANOVAs, with maternal sucrose exposure and exercise as factors. Simple effects analyses were conducted to determine the nature of significant interaction effects. Time was included as a within-subjects factor for body weight analysis. Data from the Added group, (which received access to chow and water only), Sucrose-Active and Sucrose-Sedentary groups were compared using planned contrasts. Data are presented as means ± standard errors of the mean (SEM).

## Results

### 3.1 Stage 1: Females and their offspring through weaning.

**Fig 1:**
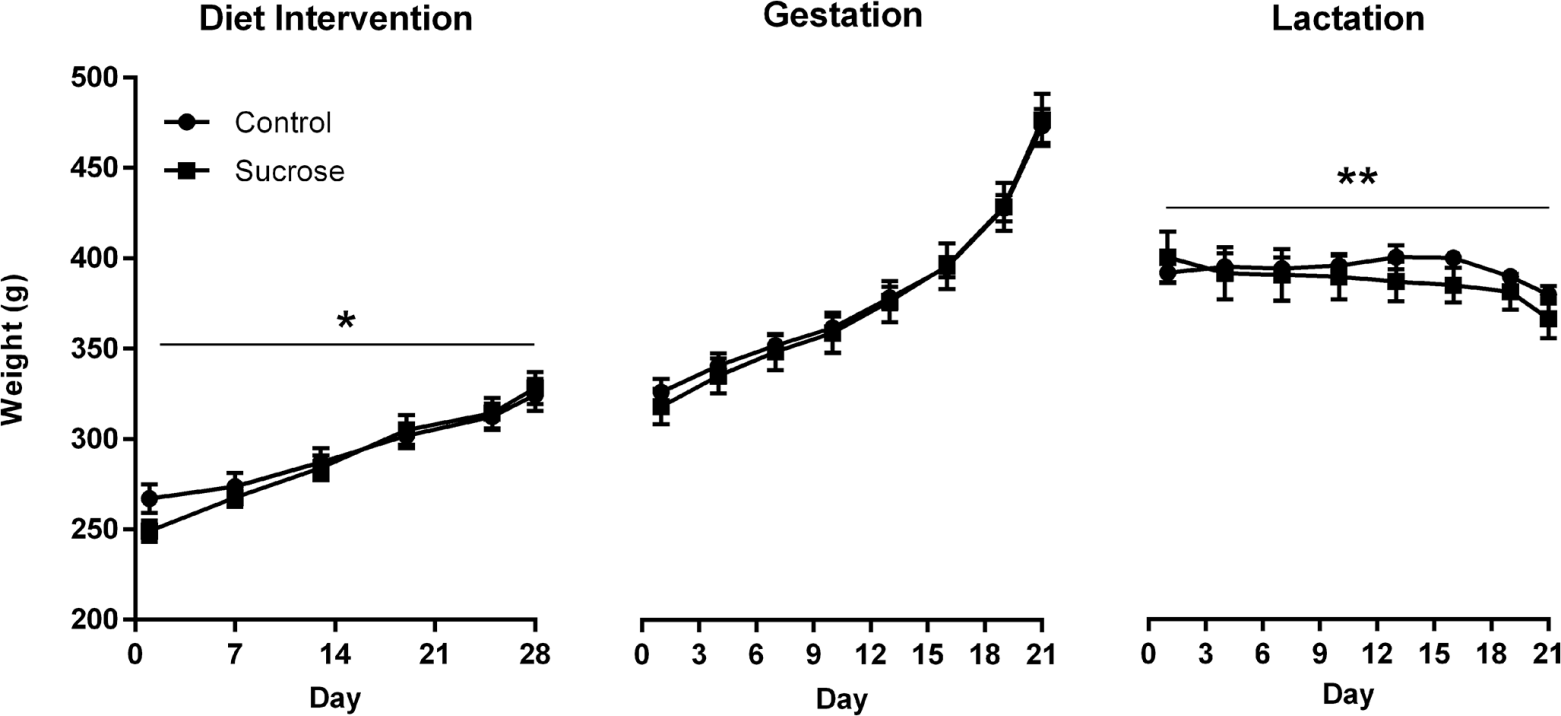
Body weights of female rats across the diet intervention, gestation, and lactation. Rats in the Sucrose group had free access to 10% sucrose solution during the 28-day diet intervention and during gestation, with access ceasing at parturition (P). Data are group means ± SEM, with *n* = 12 for the diet intervention phase and *n* = 11 for gestation and lactation phases (1 rat from each group miscarried). Relative to Controls, the Sucrose group gained a greater proportion of starting body weight during the diet intervention, *p = .02, and lost a greater proportion of weight during lactation, **p = .003. Groups did not differ in the rate of weight gain during gestation.

Body weights during the diet intervention and subsequent gestation and lactation periods are shown in Fig 1. Weights significantly increased during the 28-day diet intervention (*F*(1, 22) = 183.9, p < .0001) and there was a non-significant trend toward a time by group interaction (*F*(1, 22) = 3.75, p = .066), suggesting that the rate of increase differed between Sucrose and Control groups. Groups did not differ significantly in absolute weights at any point (largest *t*(22) = 1.82, p = 0.08, on day 1). For a more sensitive measure of body weight change we also calculated the percentage of starting body weight gained over the 28 days of the intervention. Analysis of this measure found that the Sucrose group gained a significantly greater proportion of starting body weight (32.2 ± 3.3% [SEM]) than the Control group (21.8 ± 2.5%; *t*(22) = 2.50, p = .02). During gestation weight gain significantly increased (*F*(1, 20) = 784.86, p < .0001) and the rate of increase did not differ between groups (time by group interaction: *F*(1, 20) = 1.10, p = .307). From day 1–21 of lactation, body weights declined in all females (*F*(1, 20) = 14.21, p = .001) and there was a non-significant trend towards a time by group interaction (*F*(1, 20) = 3.90, p = .062), suggesting that weight change over this interval differed between groups. The Sucrose group lost a significantly greater proportion of weight over this interval than Controls (*t*(20) = 3.31, p = .003).

**Fig 2:**
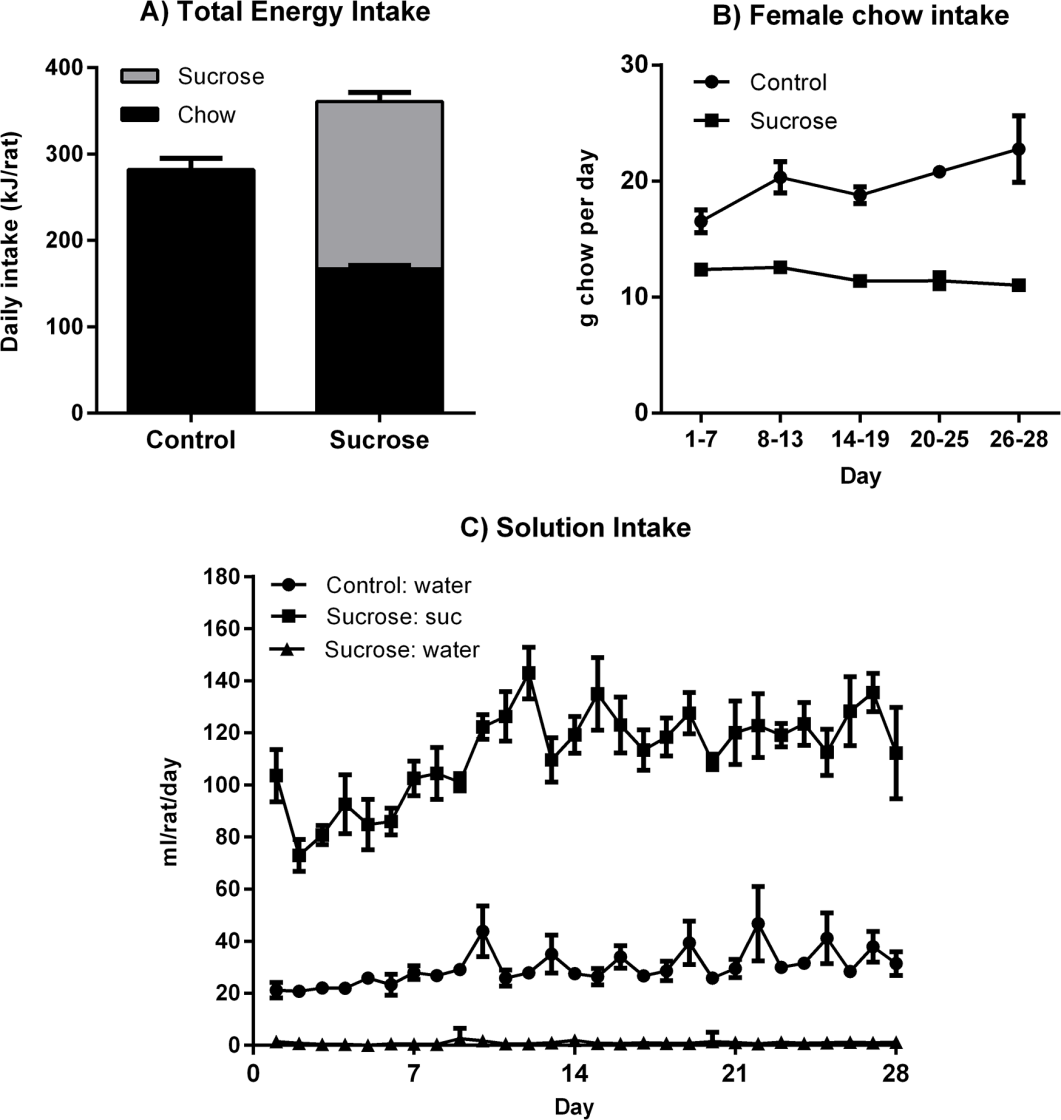
Consumption data during maternal sucrose feeding. Female rats with free access to 10% sucrose solution consumed more calories than water-only Controls (Panel A) and compensated partially for liquid calories by eating less chow (Panel B). The Sucrose group drank sucrose almost exclusively and their liquid intakes were substantially higher than water intakes in Controls (Panel C). **p < .01.

As shown in Fig 2A, during the 28 days prior to the glucose tolerance test the Sucrose rats obtained more than half of their total energy from sucrose solution and consumed 28% more total energy than the Control group (*F*(1, 4) = 27.72, p = .006). Of interest, the proportion of energy derived from sucrose solution in the Sucrose group, 53.7%, was higher than in previous experiments from our lab using male albino Wistar rats (between 36–42%) (23, 24) and we have only observed proportions above 50% in two past experiments using male Hooded Wistars (6, 23). The Sucrose group partially compensated for the energy provided by the sucrose by eating less chow than the Control group. As seen in Fig 2B, this difference in chow consumption was apparent within the first few days and persisted throughout the intervention. Analysis of chow intakes confirmed a main effect of group (*F*(1,10) = 23.25, p = 0.001), an interaction between group and linear trend (*F*(1, 4) = 8.83, p = .041) but no overall linear trend (*F*(1, 4) = 2.59, p = .183). The interaction indicated a greater increase in chow consumption in the Control than in the Sucrose group, as evident in Fig 2B. The Sucrose group consistently drank large amounts of the sucrose solution and hardly any water; their sucrose intakes were substantially higher than the Control group’s water intakes (see Fig 2C). During gestation, chow intake increased at a similar rate for Sucrose and Control females and sucrose solution intake was stable in the Sucrose group, meaning the difference in total energy intake was preserved.

**Fig 3:**
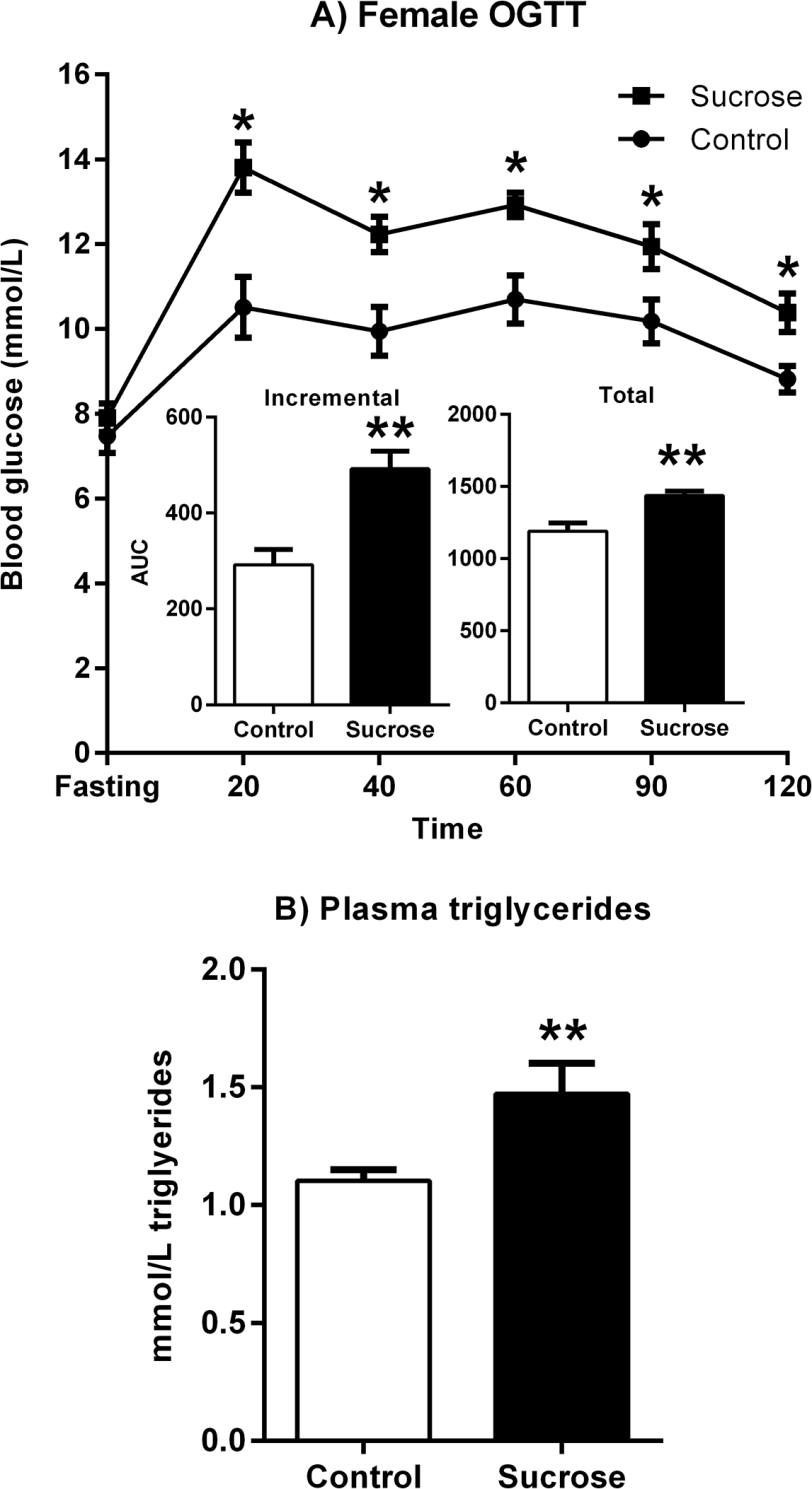
Maternal metabolic measures after sucrose feeding. Sucrose-fed female rats were glucose intolerant after 28 days of access to 10% sucrose solution, as indicated by higher incremental and total AUC during an OGTT (Panel A). Fasting blood triglycerides were significantly higher in the Sucrose group (Panel B). Data are group means ± SEM. * p < .05, ** p < .01

The most important data from Stage 1 were obtained from the OGTT and blood triglyceride tests performed on Day 29. Fig 3A shows the change in BGLs over the duration of the OGTT. The increase in blood glucose following the oral glucose load was greater in the Sucrose than in the Control group. Blood glucose peaked 20 min following gavage and remained higher in the Sucrose group relative to the Control group throughout the test. This description was confirmed by statistical analysis showing a significant increase in BGL over time (linear trend: *F*(1,22) = 8.46, p = 0.008) and a significant group by time quadratic trend (*F*(1,22) = 6.134, p = 0.021). The incremental and total Area Under the Curve (AUC) of BGLs over the OGTT were each significantly higher in the Sucrose group (*F*(1, 22) = 17.15, p < .001 and *F*(1, 22) = 14.05, p = .001, respectively–see inset, Fig 3A). Although there were no differences in fasting BGLs between Sucrose and Control groups (*t* < 1), fasting blood triglycerides were higher in the Sucrose group than in the Control group, as shown in Fig 3B (*t*(22) = 2.64, p = 0.015). Together, these data indicate that four weeks of free access to 10% sucrose produced glucose intolerance and hypertriglyceridemia in female rats prior to mating and gestation.

These female rats were culled as soon as possible after their offspring were weaned at post-natal day 21, and retroperitoneal fat pads were excised, weighed, and expressed relative to body weight. Mean ratios of g/kg retroperitoneal fat were 10.88 ± .93 (SEM) for the Control group and 11.04 ± .86 (SEM) for the Sucrose group; this difference was not statistically significant (*t* < 1).

Eleven out of the 12 females in the Sucrose group and 11 of the 12 females in the Control group delivered litters (one rat from each group miscarried). Two Control females failed to conceive on their first mating attempt but delivered normal litters after re-mating. The 11 Sucrose mothers delivered a total of 121 pups (60 females) while the 11 Control mothers delivered 133 pups (72 females). Average litter size for Sucrose mothers was 11.0 ± .96 [SEM] (range: 7–16, mean M:F ratio 5.55: 5.45) and for Control mothers was 12.09 ± .92 (range: 5–16, mean M:F ratio 5.55: 6.55). Mean litter size and sex ratio per litter did not significantly differ between Sucrose and Control groups (both *t*s < 1).

Weights on post-natal day (PND) 1 did not significantly differ between Sucrose and Control male (*t* < 1) or female offspring (*t*(18) = 1.13, p = .274). In the analyses of offspring body weight that follow each litter was represented with its mean male and female weight at each time point. Data were excluded from one Sucrose and one Control litter in which only 5 offspring survived. Thus, offspring weight analyses had *n* = 10 litters per group.

**Fig 4:**
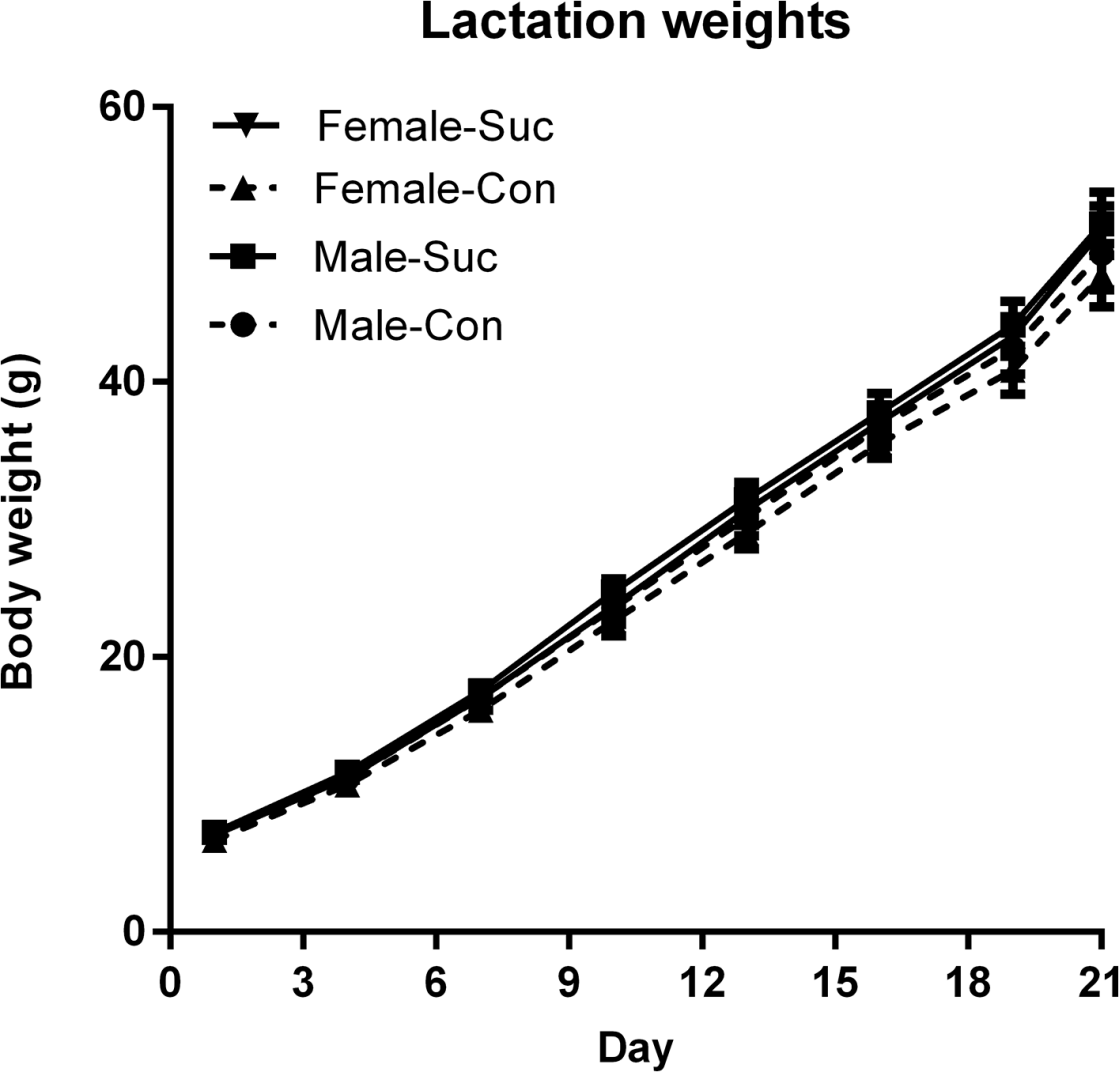
Offspring body weight gain. The rate of weight gain over lactation did not differ between offspring of Sucrose and Control mothers for either males or females.

Body weight gain from parturition to weaning is shown in Fig 4 and was analyzed in 2 x (8) mixed-ANOVAS for male and female offspring separately. These analyses showed that weight significantly increased over time for both males (*F*(1, 18) = 863.86, p < .0001) and females (*F*(1, 18) = 1157.25, p < .0001) but that this increase did not vary between offspring of Sucrose versus Control mothers (both *F*(1, 18) < 1.16). Body weights at weaning were analyzed using separate *t*-tests for males and females; there were no group differences for males (*t* < 1) or females (*t*(18) = 1.12, p = .279).

In summary, the main results from Stage 1 of this study were, first, that access to 10% sucrose solution for 4 weeks produced glucose intolerance and increased blood triglyceride levels in female rats but, second, this had no detectable effect on the size or weights of their litters and no detectable effect on the growth of their offspring.

### 3.2 Stage 2: Voluntary exercise and glucose tolerance in young adult offspring

**Fig 5:**
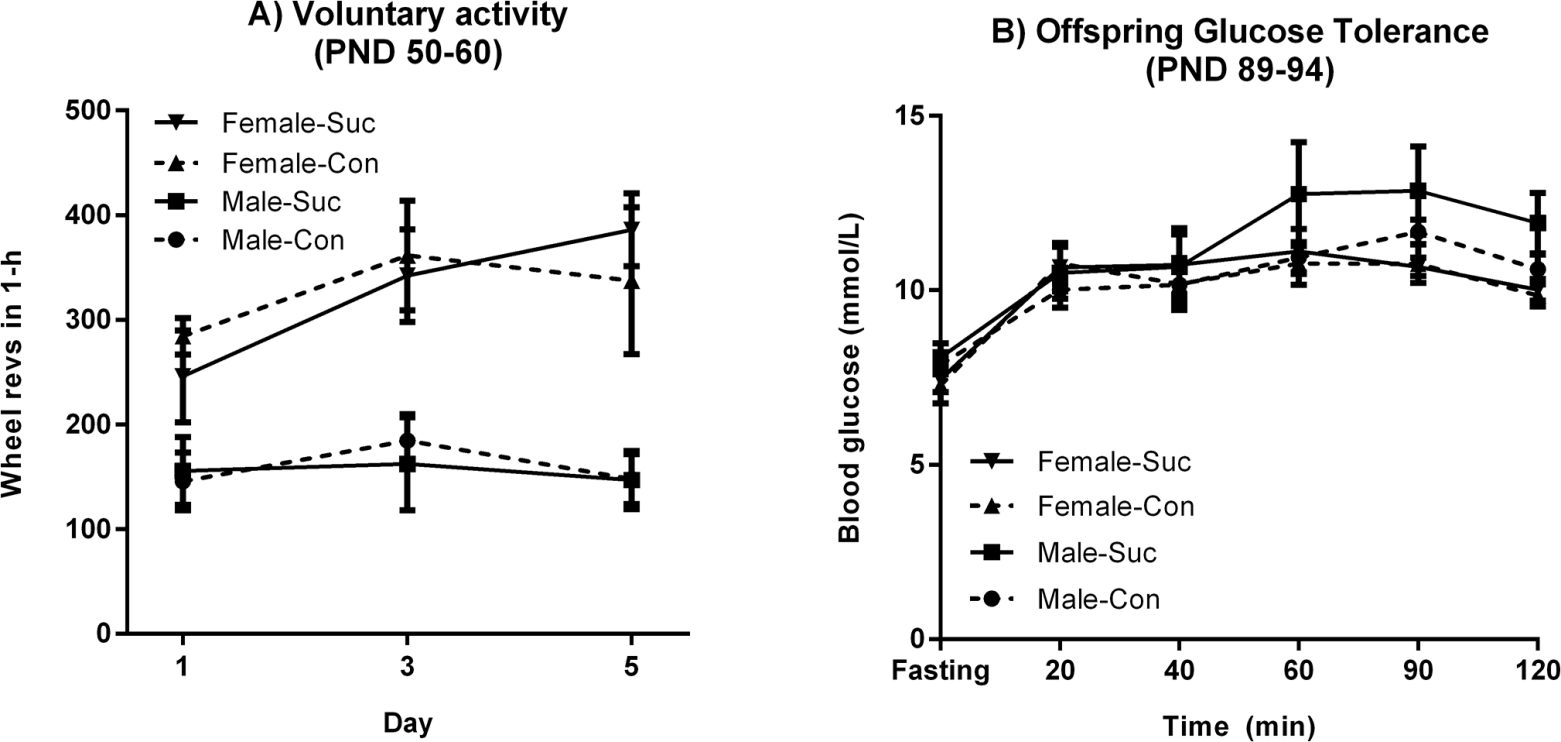
Results from Stage 2. Panel A: Voluntary activity increased over sessions in females but not in males, and there was no effect of maternal sucrose consumption. Panel B: Blood glucose levels rose more rapidly in males than in females in an OGTT, but maternal sucrose exposure had no effect.

Voluntary exercise at PND 50–60, as measured by total wheel revolutions over 1-h, is shown in Fig 5A and was analyzed in a 2 x 2 x (3) mixed-ANOVA. Wheel running significantly increased from days 1–3 (*F*(1, 34) = 5.04, p = .031) and this increase was significantly greater in females than in males (*F*(1, 34) = 5.75, p = .022) but did not differ according to maternal sucrose consumption (*F* < 1) nor an interaction between sex and maternal sucrose (*F*(1, 34) = 1.39, p > .05). Similarly, when averaging over days females ran more than males (*F*(1, 34) = 27.33, p < .001) with no effect of maternal sucrose and no interaction between sex and maternal sucrose (both *F* < 1).

Data from the OGTT (PND 89–94) are shown in Fig 5B and were analyzed using a 2 x 2 x (6) mixed-ANOVA. Blood glucose levels rose more rapidly in males than in females (sex by time linear interaction trend: *F*(1, 21) = 6.16, p = .022), but no other effects in this analysis or in 2 x 2 ANOVAs of the incremental and total AUC were significant (largest *F*(1, 21) = 1.15). Similarly, analysis of fasting glucose levels showed no effects of maternal sucrose (*F* < 1), sex (*F*(1, 21) = 2.40, p = .136) or their interaction (*F* < 1). Finally, body weights for the 40 males that were selected for Stage 3 were entered into a 2 x 2 x (7) mixed-ANOVA spanning PND 49–94. This analysis showed that the increase in weights over time (*F*(1, 34) = 1897.93, p < .0001) did not interact with maternal status (*F*(1, 36) = 1.62, p = .21). Similarly, there were no differences in body weight between Sucrose and Control female offspring at 7–8 weeks of age (*F* < 1).

In summary, Stage 2, when the offspring were 2–3 months of age, confirmed well-documented sex differences but found no detectable effects of maternal sucrose consumption on physical activity, metabolic measures or body weight gain.

### 3.3 Stage 3: Adult male offspring (PND 91–142) with 10% sucrose added to their diet.

One rat in the Sucrose-Sedentary group died from unknown causes in the third week of the intervention and its data were excluded from analyses. Because rats were group housed with only two cages per group, there was limited statistical power to detect group differences in intakes. Thus, although such an analysis found that sucrose intakes increased over time at a decreasing rate (linear trend: *F*(1, 4) = 12.89, p = .023; quadratic trend: *F*(1, 4) = 25.46, p = .007), the rate of increase did not differ according to maternal status (*F*(1, 4) = 2.11, p = .22), exercise, or their interaction (both *F* < 1). There were no significant main effects of exercise or of maternal status on sucrose consumption (*F*(1, 4) = 5.12, p = .086 and (*F*(1, 4) = 4.82, p = .082). Chow consumption per cage decreased over the intervention at a slowing rate (linear trend: *F*(1, 4) = 51.6, p = .002; quadratic trend: *F*(1, 4) = 14.29, p = .019) but again no effects of maternal status, exercise, or their interaction were detected (all *F*s < 1). As a result of their slightly higher sucrose intakes yet equal chow consumption, Sedentary groups gained a greater proportion of energy from sucrose, 42.5%, than did Active groups, 38.4% (*F*(1, 4) = 8.03, p = .047). There was a non-significant trend toward a difference between the proportion of energy derived from sucrose in cages of offspring from sucrose mothers, 38.5%, compared with cages of offspring from control mothers, 42.3% (*F*(1, 4) = 6.87, p = .059). Total caloric intake did not significantly change over the 48-day intervention (linear trend: *F*(1, 4) = 1.30, p = .319) and did not differ according to maternal diet, exercise, or their interaction (largest *F*(1, 4) = 2.90, p = .164).

**Fig 6:**
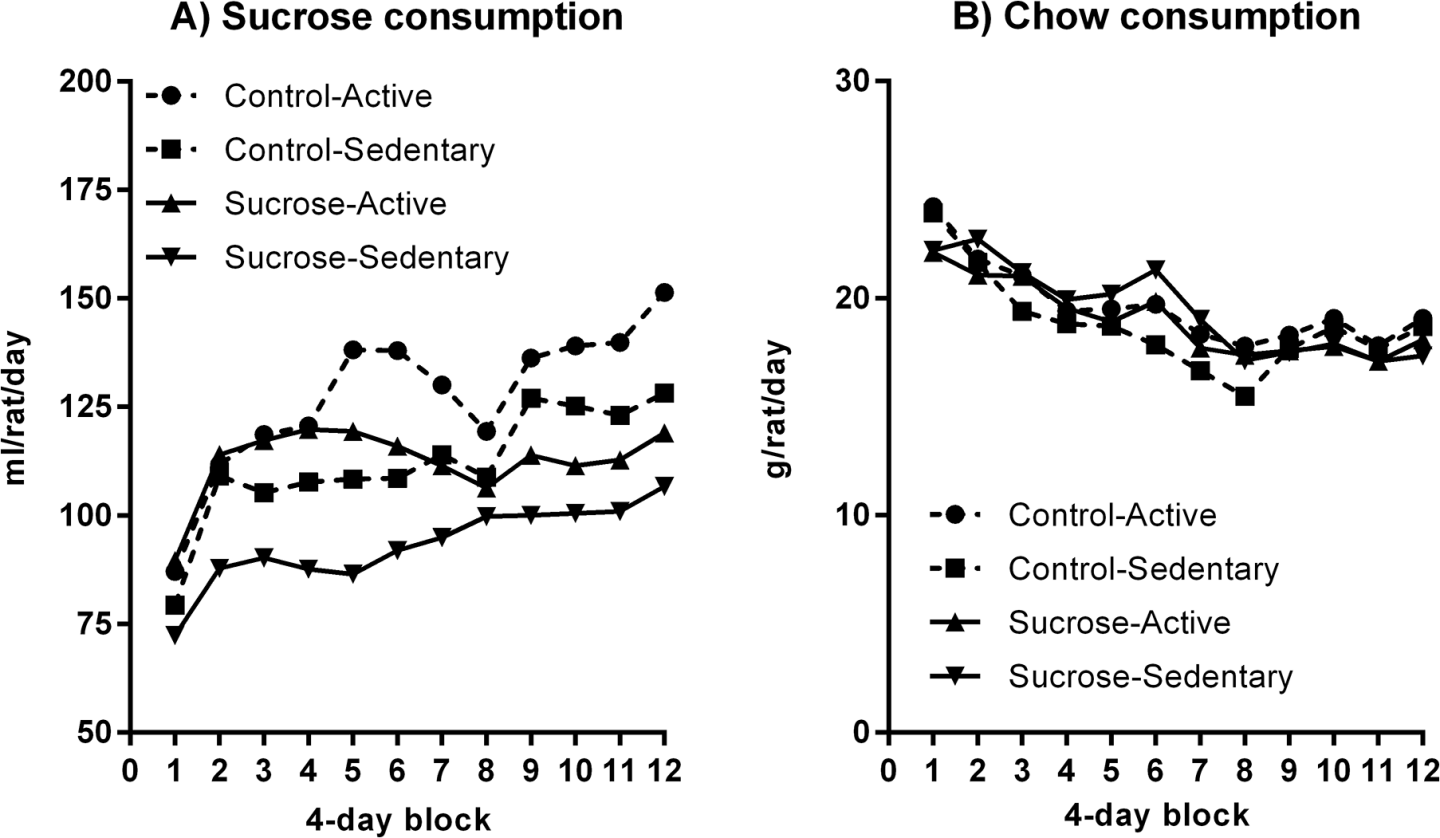
Wheel running in Stage 3. Wheel running (turns per 2-h session) did not differ between offspring of control and sucrose-fed mothers, and did not significantly change over the course of the intervention.

Fig 6 shows the mean number of wheel revolutions made by the Sucrose-Active and Control-Active groups during their 2-h sessions every second day. Although this graph suggests that the Sucrose-Active group tended to run more than the Control-Active group, this difference was not significant (*F*(1, 18) = 1.26, p = .276) and there was no significant change in activity levels over time (linear trend: *F* < 1).

**Fig 7:**
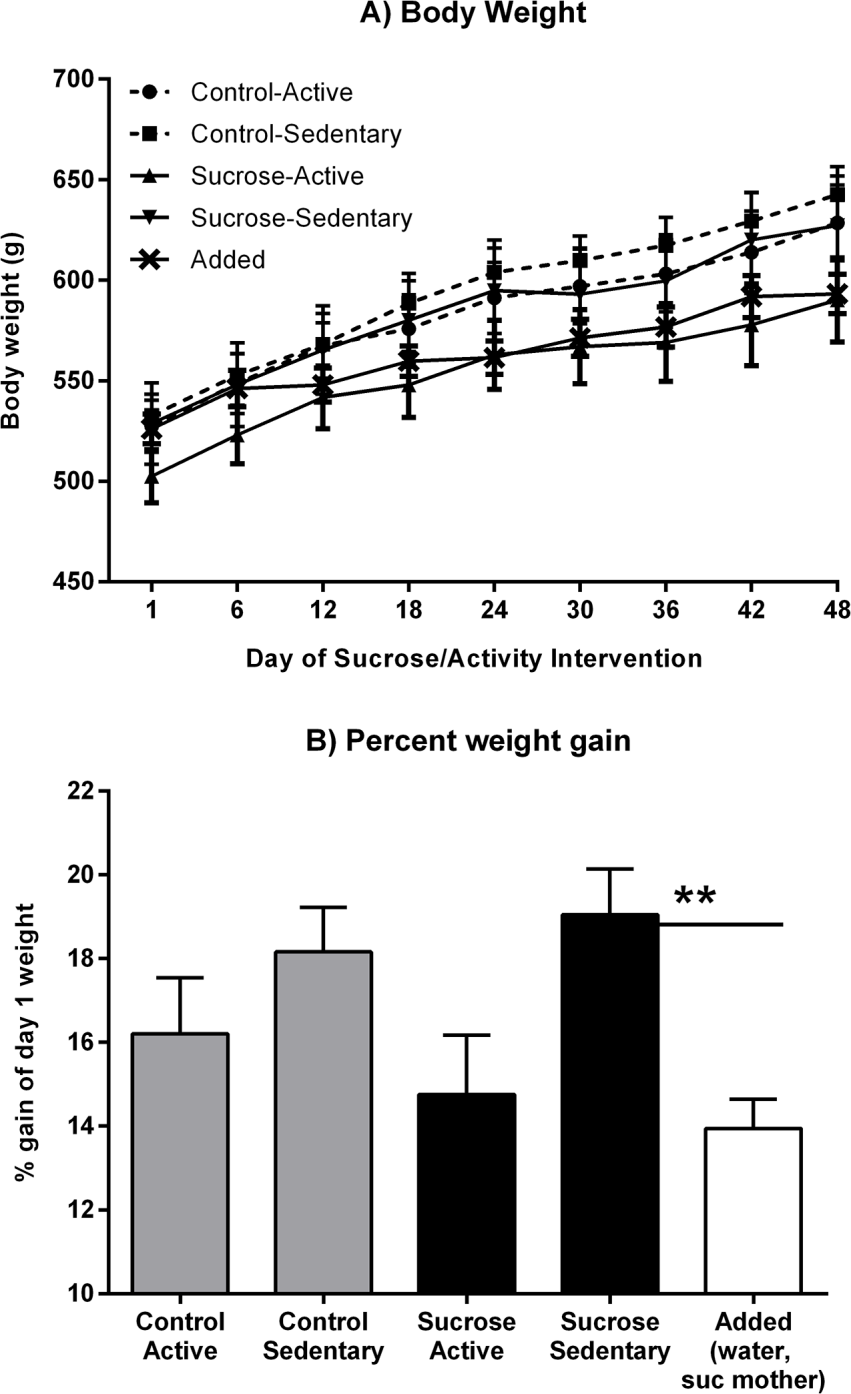
Body weight gain (A) and percent change (B) over the 48-day sucrose and activity intervention. Active groups gained a smaller proportion of starting body weight than Sedentary groups. Within offspring of sucrose mothers, the Sucrose-Sedentary group gained more weight than Added group not given sucrose, whereas no difference was detected between the latter and the Sucrose-Active group. ** p = .003.

Body weights and percent weight change are shown in Fig 7A and 7B. Weights increased over the 7 weeks of the intervention (*F*(1, 35) = 456.52, p < .001) with a non-significant trend towards slower weight gain in Active than in Sedentary groups (*F*(1, 35) = 3.22, p = .081). However, no effect of maternal diet was detected nor any interaction between this factor and exercise (largest *F*(1, 35) = 1.64, p = .209; see Fig 7A). When percent weight change over the intervention was analyzed, a 2 x 2 ANOVA showed that this was smaller in Active groups than in Sedentary groups (*F*(1, 35) = 7.51, p = .010) but neither an effect of maternal diet nor an interaction between the two factors was detected (largest *F*(1, 35) = 1.35, p = .25; Fig 7B). The percent weight gain by the Added group that had only chow and water access throughout the intervention was lower than the gain by the Sucrose-Sedentary group (*F*(1, 42) = 10.33, p = .003) but did not differ from the gain by the Sucrose-Active group (*F* < 1).

The results of the memory tests given to the four main groups showed no main or interaction effects of exercise or maternal diet on exploration time or exploration ratios in either the baseline or midway tests of object or place recognition memory (largest *F*(1, 34) = 2.32, p = .137). The one result of interest was an overall effect of test type (*F*(1, 33) = 43.59, p < .001) indicating that place recognition memory was significantly poorer than object memory. This result did not interact with time or group factors (all *F* < 1).

**Fig 8:**
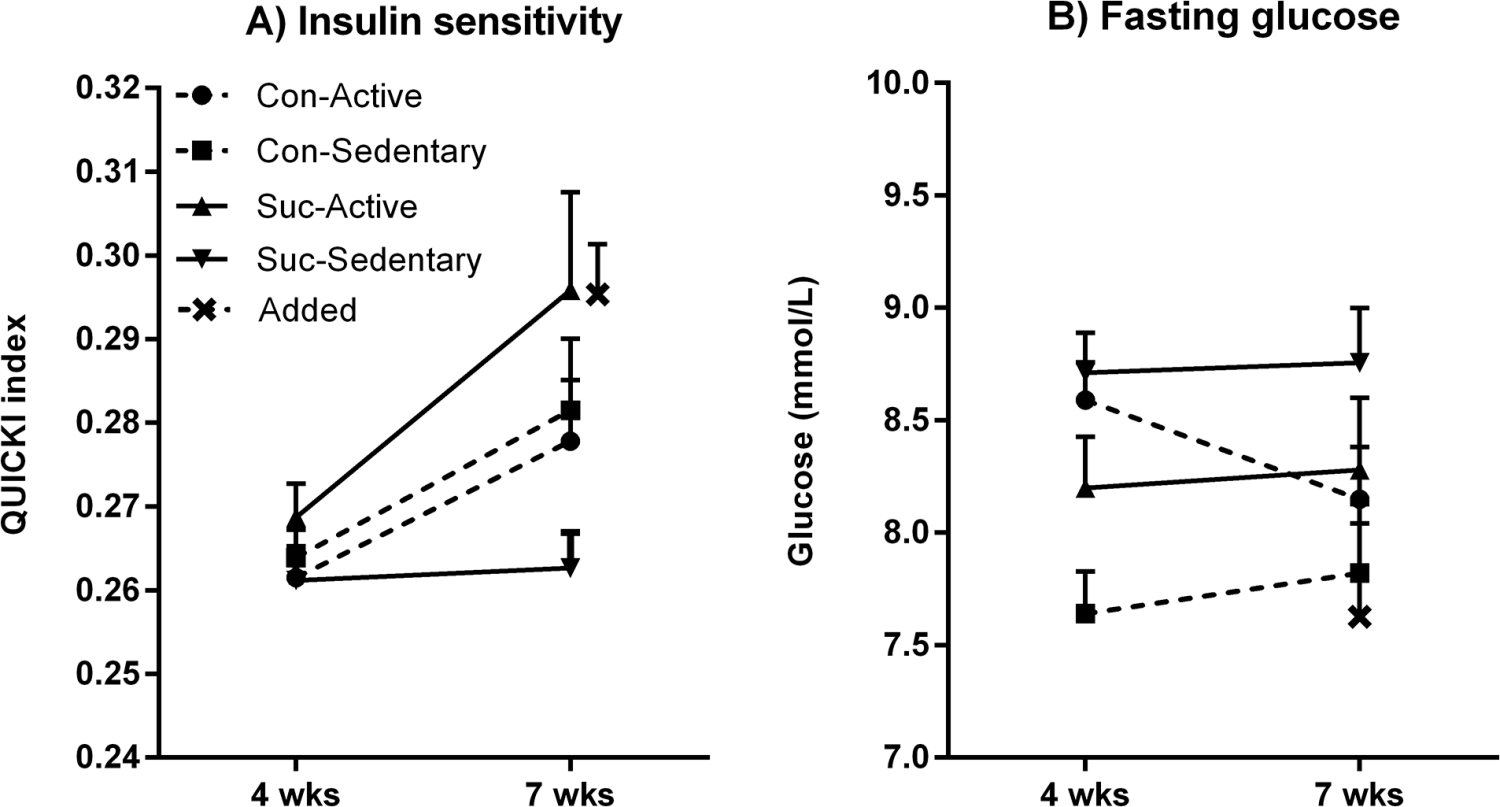
Metabolic measures in Stage 3. Panel A: At 7 weeks insulin sensitivity was increased by activity in the offspring of sucrose-fed mothers (Sucrose-Active *vs*. Sucrose-Sedentary groups) but no such effect was detected in the offspring of control mothers (Control-Active *vs*. Control-Sedentary groups). Panel B: Fasting blood glucose levels were higher overall in the offspring of sucrose-fed mothers than in offspring of control mothers, and when the Added group was measured at 7 weeks its levels were lower than those of the Sucrose-Sedentary group but not significantly different from those of the Sucrose-Active group. **p = .01.

As in Stage 1, the most important data from Stage 3 were those obtained from the metabolic measures. Insulin sensitivity was calculated using the QUICKI transformation (QUICKI = 1/(log[Gluc] + log[Ins])) and is displayed in Fig 8A. While no group differences in insulin sensitivity were detected following 4 weeks of access to sucrose (all *F*s < 1), insulin sensitivity rose significantly from weeks 4 to 7 (*F*(1, 27) = 18.52, p < .001) and this rise varied according to an interaction between exercise and maternal status (*F*(1, 27) = 7.31, p = .012). When a subsequent 2 x 2 ANOVA was applied to the week 7 measures, there were no differences in insulin sensitivity between active and sedentary groups (*F*(1, 32) = 3.21, p = .083) but there was a significant interaction between activity and maternal diet (*F*(1, 32) = 5.01, p = .032). Simple effects analyses showed that exercise significantly increased insulin sensitivity in offspring of sucrose-fed mothers (*F*(1, 32) = 7.69, p < .01) but no such effect was found in offspring of control mothers (*F* < 1, see Fig 8A). Planned contrasts showed that insulin sensitivity in the Added group was significantly higher than in the Sucrose-Sedentary group (*F*(1, 39) = 8.25, p = .007) but did not differ from the Sucrose-Active group (*F* < 1). This pattern of results resembles that obtained when similar contrasts were applied to percent weight gain, as reported above.

Fasting blood glucose levels (BGLs) after 4 and 7 weeks of sucrose access are shown in Fig 8B. A 2 x 2 x (2) mixed-design ANOVA applied to these data found that on average, BGLs were higher in the offspring of sucrose-fed mothers than in control offspring (*F*(1, 35) = 4.72, p = .037) and there was an interaction between exercise and maternal diet(*F*(1, 35) = 7.94, p = .008) but no main effect of activity (*F* < 1). The nature of this interaction was examined using tests for simple effects, which showed a significant difference between control and sucrose offspring that were sedentary (*F*(1, 35) = 12.12, p = .001) but not between active control and active sucrose offspring (*F* < 1). Fasting BGLs did not significantly change from week 4–7 and change over time did not interact with any group factors (largest *F*(1, 35) = 1.50, p = .229). At week 7, BGLs in the Added group were significantly lower than those in the Sucrose-Sedentary group (*F*(1, 42) = 6.19, p = .017) but did not differ from the Sucrose-Active group (*F*(1, 42) = 2.18, p = .147).

**Fig 9:**
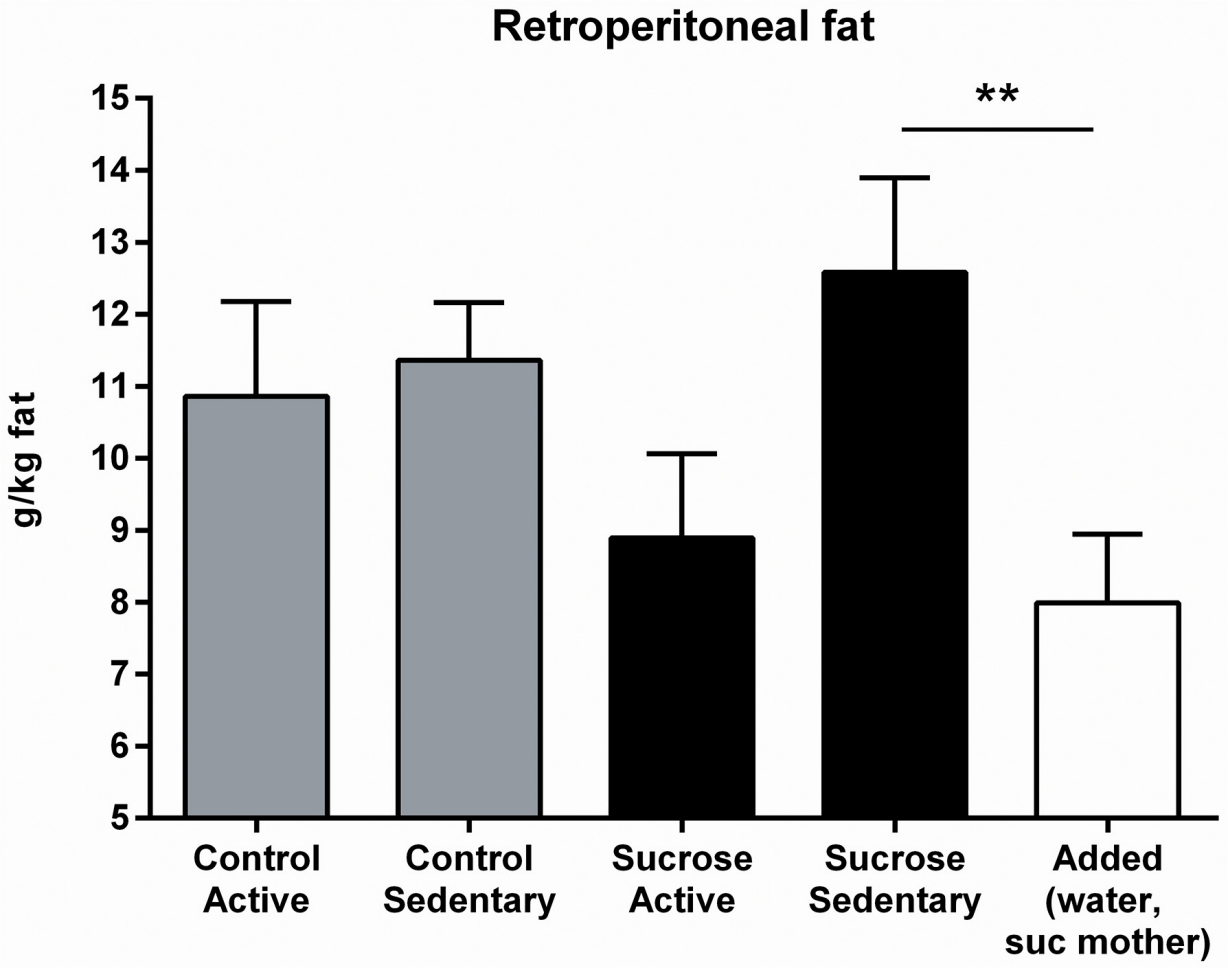
Retroperitoneal fat in Stage 3. Fat pad mass did not differ between the four experimental groups, but was lower in the Added group than in the Sucrose-Sedentary group.

Retroperitoneal fat pad mass, expressed as g/kg total body weight, is shown in Fig 9. There was a non-significant trend toward lower fat pad mass in Active than in Sedentary groups (*F*(1, 35) = 3.24, p = .08) but neither an effect of maternal diet (*F* < 1) nor an interaction effect was found (*F*(1, 35) = 1.87, p = .181). Planned contrasts found that fat pad mass in the Added group was significantly lower than in the Sucrose-Sedentary group (*F*(1, 42) = 7.30, p = .01) but did not differ from the Sucrose-Active group (*F* < 1).

## Discussion

The major results from this study were as follows. In Stage 1, giving female rats unrestricted access to 10% sucrose in addition to chow and water for four weeks produced glucose intolerance and elevated blood triglycerides (see Fig 3), even though minimal impact on body weight was detected. Despite continued access to the sucrose solution during the further three weeks or so until these females gave birth, no difference was observed between their offspring and those of control mothers in terms of litter size, birth weights or growth rates during weaning. In Stage 2, when the offspring were young adults, on measures of body weight, glucose tolerance and voluntary wheel running there were no differences between offspring of sucrose and control mothers, although sex differences in body weight and in running in activity wheels were observed, consistent with previous reports.

It was only in Stage 3, when male offspring were given unrestricted access to 10% sucrose solution with or without activity, that metabolic effects of maternal diet were detected. These consisted of increased insulin sensitivity produced by exercise in the offspring of sucrose-fed mothers (see Fig 8A), whereas no effect of exercise on these measures was detected in control offspring, and higher fasting blood glucose levels in the offspring of sucrose-fed mothers than in the offspring of control mothers (see Fig 8B). On the other hand behavioural measures in this stage–wheel running, object and place recognition memory–did not reveal any influence of maternal diet.

The results of Stage 1 indicated that four weeks of access to 10% sucrose solution produced similar effects in female rats to those we have previously reported in male rats [6, 9, 23–25]. These results are noteworthy in that the duration of access and concentration of sucrose are each less than in a majority of past research. On the other hand the failure to find any initial effect on their offspring was unexpected. As noted in the Introduction, whereas a large number of studies have reported maternal diet effects where mothers were given various kinds of high fat diets [12], few published studies have looked for such effects following an experimental diet consisting only of added sugar. Of the latter, apparently only one used sugar concentrations similar to that used in the present study. In that study [20], the experimental group of greatest interest here consisted of pregnant rats given 10% sucrose solution and then switched to chow only when their pups were born. At PND15 the female offspring of these sugar-fed mothers weighed less than offspring of chow-only control females, whereas the male offspring of sugar-fed mothers weighed more than male controls.

One possible reason for the absence of weight differences between pups in Stage 1 of the present study is the use of Wistars instead of Sprague-Dawleys [17]. A second possibility arises from differences in when sucrose was first introduced. In the present study female rats had access to sucrose for over 7 weeks before giving birth; in the experiment by Bocarsly et al. [20], access to sugar commenced on the fifth day of gestation. The three reports of increased body weight gain in the offspring of sugar-fed mothers have been from studies where sucrose was not introduced until gestation [18, 20, 22]. Only one reported study has manipulated the time at which sucrose- or starch-containing diets were introduced; namely for 25, 65 or 110 days prior to parturition. This experiment found no differences in foetal weights between Sucrose and Control dams at any duration [17], but dams fed the sucrose diet for 110 days developed elevated plasma triglycerides. Thus, our study and this previous report [17] each observed sucrose-induced hypertriglyceridemia in females with no effects on offspring body weight, despite commencing sucrose access weeks prior to mating. However, another study in mice that commenced access to sucrose 6 weeks prior to mating did find increased body weight gain in offspring of sucrose mothers [14]. Nonetheless, these data point to the importance of establishing more precisely how the duration of access to sucrose prior to mating determines offspring metabolic health.

The results from Stage 3 of the present study indicate that some effects of the mother’s sucrose consumption can be detected when their offspring reach adulthood and are themselves given access to sucrose solution. Since younger rats can be more greatly affected than older rats by the introduction of sugar drinks [25, 27–29], it seems likely that more substantial effects of the mothers’ diet than those found in Stage 3 could have been detected if the offspring had been introduced to sucrose at a younger age.

In Stage 3 the Added group consisted of male offspring of sucrose-fed mothers that were maintained on chow and water throughout this phase. Data from the Added group are of some interest in that, when compared to the Sucrose-Active and Sucrose-Sedentary groups, they suggest that the exercise protocol almost completely cancelled out the effects of sucrose consumption in terms of weight gain (see Fig 7), insulin sensitivity (see Fig 8) and retroperitoneal fat (see Fig 9). This is consistent with previous evidence we have obtained for the effect of exercise on sucrose-fed rats. In that previous study [6] the low-volume exercise protocol– 20-min treadmill training every 3 days–protected rats from sucrose-induced metabolic disturbance when measured after the first 28 days, but these beneficial effects were no longer detected after 56 days. In the present study the protective value of the more frequent and longer-duration exercise protocol– 2-h running every other day–persisted for 48 days. However, caution is needed in drawing conclusions from comparisons involving the Added group, since these rats did not undergo the behavioural testing given to the four main groups in Stage 3.

The lack of group differences on the behavioural measure of spatial location memory is inconsistent with the recent results of a recent study [22] which reported deficits in the Morris Water Maze in male offspring of female rats fed 20% sucrose solution throughout pregnancy. However, rats in that study were tested in the absence of sucrose, whereas in the present experiment testing was held after 7 weeks’ consumption of 10% sucrose. Our dietary intervention may have obscured subtle maternal effects by impairing spatial memory in all groups; this possibility is supported by the observation that performance was poorer overall in the place task, as noted earlier. It appears unlikely that the different task requirements of the place/object (land-based exploration) and Morris Water Maze (water-based escape) paradigms explain the inconsistent results, since we have previously observed deficits in both tasks in sucrose-fed rats (although in different experiments) [24, 25].

In summary, in contrast to the substantial effects on offspring of maternal diets that contain 65% sucrose [15]; 50% fructose or 50% sucrose [16]; sweetened-condensed milk [14] or 20% sucrose solution [22], in the present study feeding female rats 10% sucrose produced only limited and subtle effects. This was despite the females’ development of glucose intolerance and hypertriglyceridemia after four weeks of sucrose consumption prior to pregnancy. As noted, the previous study to have examined the effects of a 10% sucrose addition to the maternal diet found larger effects on offspring than in the present study, despite providing sucrose only when the rats were already pregnant [20]. The intriguing possibility that this is the important procedural difference between the two studies merits investigation.
